# Separating Movement and Gravity Components in an Acceleration Signal and Implications for the Assessment of Human Daily Physical Activity

**DOI:** 10.1371/journal.pone.0061691

**Published:** 2013-04-23

**Authors:** Vincent T. van Hees, Lukas Gorzelniak, Emmanuel Carlos Dean León, Martin Eder, Marcelo Pias, Salman Taherian, Ulf Ekelund, Frida Renström, Paul W. Franks, Alexander Horsch, Søren Brage

**Affiliations:** 1 Medical Research Council Epidemiology Unit, Institute of Metabolic Science, Cambridge, United Kingdom; 2 MoveLab, Institute of Cellular Medicine, Newcastle University, Newcastle Upon Tyne, United Kingdom; 3 Institute for Medical Statistics and Epidemiology, Klinikum rechts der Isar der TU München, Munich, Germany; 4 Fakultät für Informatik, TU München, Munich, Germany; 5 Computer Laboratory, Cambridge University, Cambridge, United Kingdom; 6 Department of Sport Medicine, Norwegian School of Sport Sciences, Oslo, Norway; 7 Genetic Epidemiology and Clinical Research Group, Department of Public Health and Clinical Medicine, Section for Medicine, Umeå University Hospital, Umeå, Sweden; 8 Department of Clinical Sciences, Genetic and Molecular Epidemiology Unit, Lund University, Malmö, Sweden; Wageningen University, The Netherlands

## Abstract

**Introduction:**

Human body acceleration is often used as an indicator of daily physical activity in epidemiological research. Raw acceleration signals contain three basic components: movement, gravity, and noise. Separation of these becomes increasingly difficult during rotational movements. We aimed to evaluate five different methods (metrics) of processing acceleration signals on their ability to remove the gravitational component of acceleration during standardised mechanical movements and the implications for human daily physical activity assessment.

**Methods:**

An industrial robot rotated accelerometers in the vertical plane. Radius, frequency, and angular range of motion were systematically varied. Three metrics (Euclidian norm minus one [ENMO], Euclidian norm of the high-pass filtered signals [HFEN], and HFEN plus Euclidean norm of low-pass filtered signals minus 1 g [HFEN_+_]) were derived for each experimental condition and compared against the reference acceleration (forward kinematics) of the robot arm. We then compared metrics derived from human acceleration signals from the wrist and hip in 97 adults (22–65 yr), and wrist in 63 women (20–35 yr) in whom daily activity-related energy expenditure (PAEE) was available.

**Results:**

In the robot experiment, HFEN_+_ had lowest error during (vertical plane) rotations at an oscillating frequency higher than the filter cut-off frequency while for lower frequencies ENMO performed better. In the human experiments, metrics HFEN and ENMO on hip were most discrepant (within- and between-individual explained variance of 0.90 and 0.46, respectively). ENMO, HFEN and HFEN_+_ explained 34%, 30% and 36% of the variance in daily PAEE, respectively, compared to 26% for a metric which did not attempt to remove the gravitational component (metric EN).

**Conclusion:**

In conclusion, none of the metrics as evaluated systematically outperformed all other metrics across a wide range of standardised kinematic conditions. However, choice of metric explains different degrees of variance in daily human physical activity.

## Introduction

The assessment of human daily physical activity in population studies requires accurate, cheap, and feasible measurement technology [1], [2], [3]. Accelerometers are increasingly being used for physical activity assessment and most of the accelerometers that have been used in population studies express their output in proprietary units usually referred to as “counts” [4], [5].

Accelerometer devices, based on acceleration sensors which allow for raw data storage expressed in g-units or SI units at a relatively high sampling frequency have been used in gait analysis [6], [7] and ambulant activity classification [8], [9] for a number of years. The output of raw accelerometers is not summarized by the monitor allowing for increased control over data processing by the end-user in contrast to the traditional accelerometers. Technological developments in recent years have made raw accelerometry feasible for population research, allowing weeklong data collection.

A measured acceleration signal consists of a gravitational component, a movement component, and noise [9]. During static conditions or conditions of steady state non-rotational movement, the gravitational component is visible as the offset of one or more sensor axes and can then be used for detection of the sensor orientation relative to the vertical plane [9]. The separation of the gravitational component from the acceleration signal is complicated by the fact that in the presence of rotational movements the frequency domains of the movement-related component and the gravitational component can overlap, thus making simple frequency-based filtering inappropriate for perfect separation.

The first two studies that identified the challenge of separating the components of acceleration lacked a comparison against a reference method [10], [11]. Studies by Bouten et al. and Bourke et al. used a reference method, but were limited to laboratory experiments that may not generalise to accelerometer data collected under real life conditions [12], [13]. None of the studies as mentioned above systematically evaluated how metric accuracy varies across magnitudes and frequencies of acceleration. Characterisation of the latter may be important to gain insight into metric performance under real-life conditions.

The use of gyroscopes in addition to acceleration sensors could be regarded as the solution for separating the gravitational component from the acceleration signal [14], [15], [16]. However, these devices do not yet meet feasibility requirements for use in large scale observational research. Raw accelerometry has been applied in various epidemiological studies since it became sufficiently feasible in the period 2008–2010. Most of these studies are not published yet, but already amount to over ten thousand participants. None of these datasets include gyroscopic data and therefore require an accelerometer-specific solution.

The main objective of the present study was therefore to evaluate the ability of different methods (metrics) of processing acceleration signals to remove the gravitational component of acceleration by comparison against a reference method under a range of standardised kinematic conditions. A second objective was to assess the shared variance between these metrics in human physical activity data collected during daily life and the impact of metric selection on the accuracy with which daily energy expenditure can be estimated.

## Methods

### Ethics Statement

Ethical approvals were obtained from the Cambridgeshire research ethics committee, Cambridge (UK) and from the Regional Ethical Review Board in Umeå (Sweden).

### Study Design

The main experiment in this study was done with a robot and did not involve testing of human participants. Two additional sets of experiments were performed, the first to test the degree to which metrics convey similar information when applied to wrist and hip signals, and the second to assess the implication of such differences for estimation of daily physical activity-related energy expenditure.

### Robot Experiment

**Figure 1:**
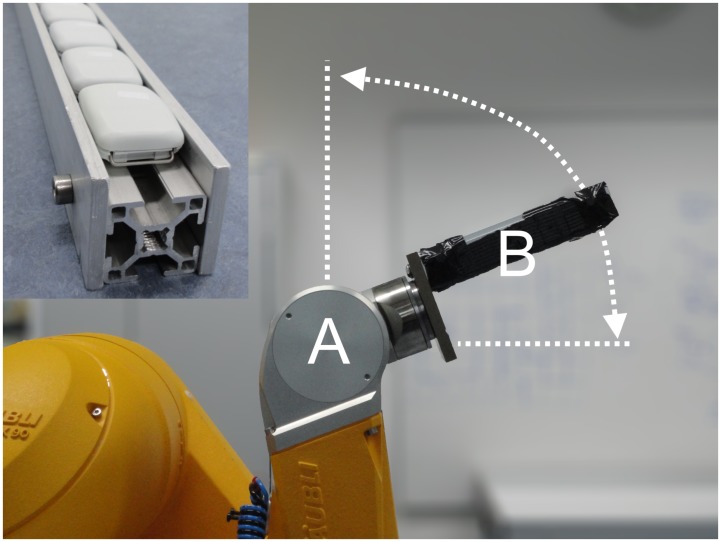
Experimental setup. A bar (B) holds five accelerometers and rotates around robot joint (A).

**Figure 2:**
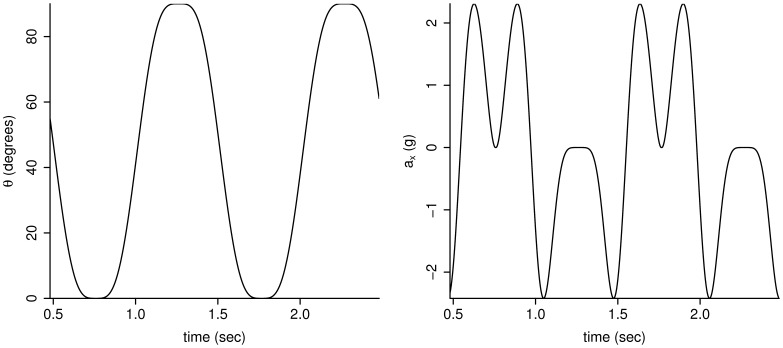
Robot joint angle and horizontal acceleration for condition: 1 Hz, amplitude 45°, radius = 0.5 m.

An industrial robot (TX90, Stäubli Tec-Systems GmbH, Bayreuth, Germany; see **Figure 1**) was used to rotate accelerometers (GENEA, Unilever Discover, Sharnbrook Bedfordshire, UK) in the vertical plane following a general minimum-jerk oscillatory motion (single plane). The motion was applied to establish a standardized alternating contribution of gravity to the accelerometer output. The robot consists of an articulated arm with six joints from which the fifth joint counted from the base of the robot was used in this study. The oscillating motion was continuous (non-damping) around a single horizontal axis. The trajectory was programmed using a 7th order polynomial function with kinematic constraints **(Supporting Information S1)**. A high order function was needed to reduce the natural vibrations transmitted between the robot and its own base [17], [18]. An example of the angular position over time for one experimental condition is given in **Figure 2**.

The frequency of oscillation, the radius of rotational movement (shortest distance to centre of rotation), and the angular range of motion were systematically varied. The range of frequency conditions was limited by the maximal amount of mass moment of inertia and torques that could be absorbed by the robot and supporting frame. For all frequencies ranging from 0.05 Hz to 1.2 Hz, eighteen tri-axial accelerometers were positioned along the length of a 70 cm bar mounted to the flange of the robot at 10 cm from the centre of rotation. The application of eighteen accelerometers in parallel allowed for assessment of the relationship between metric output and the radius of movement. To reduce mass moment of inertia at the higher frequencies of oscillation (>1.1 Hz) a shorter bar (20 cm) was used, see **Figure 1**. The shorter bar provided space for the attachment of only five accelerometers. The torque can be further reduced by reducing the range of angular rotation; some experimental conditions were defined by this constraint. For reference purposes, all eighteen accelerometers were also tested under static conditions (no robot movement) at angles 0° and 22.5°. Each experimental condition was done for three minutes. An overview of all experimental conditions is shown in **Table 1**. For monitoring potential vibrations, a source of experimental error, one additional accelerometer was attached to the base of joint 5 for all experimental conditions. The base of joint 5, i.e. the robotic with its joint 1 up to joint 4, should in theory not move during these experiments.

**Table 1:** Experimental conditions of the robot setup.

Frequencies	Angle range*	Number of accelerometers (range in position relative to axis of rotation)
0 Hz	0° and 22.5°	18 (0.13–0.78 m)
0.05 to 0.55 Hz (steps of 0.05)	0–90°	18 (0.13–0.78 m)
0.60, 0.70, and 0.80 Hz	0–45°	18 (0.13–0.78 m)
0.90, 1.00, and 1.10 Hz	0–20°	18 (0.13–0.78 m)
1.20 and 1.30 Hz	0–45°	5 (0.13–0.29 m)
1.4 to 2.6 (steps of 0.1), 2.8, 3.0, 3.2, 3.6 and 4 Hz	0–20°	5 (0.13–0.29 m)

[*for 0° the bar is in horizontal position and for 90° the bar is pointing upwards relative to the axis of rotation].

### Human Experiments

In order to facilitate the interpretation of the robot experiment in the context of human daily (free-living) physical activity, we asked 47 men and 50 women (healthy, aged 22–65 yrs) to wear accelerometers on their wrist and on their hip for seven days during free-living as previously described [19]. We also re-analysed wrist acceleration signals obtained during free-living conditions from 65 healthy women (aged 20–35 yrs) as previously described [19]. In this latter sample, physical activity-related energy expenditure (PAEE) was assessed using the doubly labelled water method in combination with resting energy expenditure measured by indirect calorimetry [19]. For both human studies, objectives and procedures were explained in detail to the participants, after which they provided written and verbal informed consent.

### Accelerometer

The accelerometer comprised a tri-axial STMicroelectronics accelerometer (LIS3LV02DL) with a dynamic range of ±6 g (1 g = 9.81 m·s^−2^), as described elsewhere [20]. The acceleration was sampled at 80 Hz and data were stored in g units for offline analyses. In the robot experiment, the accelerometer was aligned by two aluminium strips on each side of the bar (insert, **Figure 1**) and covered by duck-tape on top, see **Figure 1**. The radius length, i.e. the distance from the axis of rotation to the accelerometer chip, was assessed by measurement tape to the closest mm. The position of the accelerometer chip inside the accelerometer packaging was obtained from the manufacturer. In the human experiment, the accelerometers were attached to the wrist with a nylon weave strap and to the hip with an elastic belt. Participants were instructed to wear the accelerometer on the wrist continuously for 24 hours per day throughout the whole observation period and to remove the hip accelerometer during sleeping hours. The manufacturer calibration of all acceleration sensors was tested under static conditions (no movement, vector magnitude = 1 g) and adjusted if necessary.

### Metrics

For the robot analyses three metrics for the estimation of acceleration related to movement were evaluated: (i) the Euclidean norm (vector magnitude) of the three raw signals minus 1, referred to as ENMO; (ii) the application of a high-pass frequency filter (4^th^ order Butterworth filter with ω_0_ = 0.2 Hz) to each raw signal, after which the Euclidean norm was taken from the three resulting signals, 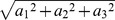, referred to as HFEN, and; (iii) metric HFEN plus the Euclidean norm of the three low-pass filtered raw signals (4^th^ order Butterworth with ω_0_ = 0.2 Hz) minus 1 g, referred to as HFEN_+_.

The third metric has not been described previously. The motivation for metric HFEN_+_ is as follows: In the absence of rotational movement the Euclidian norm of the three low-pass filtered raw signals (LFEN) is equal to 1 g. In the presence of rotation, however, LFEN may be different to 1 g due to imperfect separation; there we add this difference (positive or negative) to HFEN. A low frequency component above 1 g may result from low-frequency accelerations perpendicular to the direction of rotation, e.g. the centripetal force when sitting on a swing. A low frequency component below 1 g could indicate that part of the gravitational component is still contained in the high-frequency content, e.g. rotations in the vertical plane as a result of which gravity is an alternating component in the signal. A further elaboration on the motivation for metric HFEN_+_ can be found in **Supporting Information S1**.

For some of the metrics described above the output could in theory be negative. To gain insight into when this happens, negative values were not corrected for the robot experiment. However, for the accelerometer data collected in daily human movement, negative metric output was rounded off to zero before further analysis.

The filter cut-off frequency of 0.2 Hz for metrics HFEN and HFEN_+_ was chosen on the presumption that most of daily acceleration related to movement for most human body parts occurs at frequencies higher than 0.2 Hz. n the robot experiment, the exact absolute value of this filter cut-off frequency (0.2 Hz) was considered of minor relevance as this experiment intends to investigate frequency of rotation and frequency of filtering on a relative scale. For the human part of our study, both a cut-off frequency of 0.2 Hz and 0.5 Hz were evaluated to assess the effect of threshold selection in relation to human movement. Additionally, the human part of our study was extended with the application of a band-pass frequency filter version of HFEN (4^th^ order Butterworth filter with ω_0_ = 0.2–15 Hz), referred to as BFEN, to assess the effect of high-frequency noise removal.

Finally, the Euclidean norm of the three raw acceleration signals (EN) without subtraction of gravity was added to the evaluations in human data to assess the relevance of attempting to remove the gravitational component from an applied perspective.

To sum up, metrics evaluated in this investigation include Euclidian Norm (EN), Euclidian Norm Minus One (ENMO), Bandpass-Filtered followed by Euclidian Norm (BFEN), Highpass-Filtered followed by Euclidian Norm (HFEN), and Highpass-Filtered followed by Euclidian Norm Plus difference between 1 g and low-pass-filtered component (HFEN_+_).

### Analysis

Reference values for robot acceleration were calculated based on forward kinematics of the robot arm using the radius length (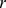) of each accelerometer relative to the axis of rotation and the robot arm’s angle 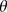, angular velocity 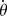, and angular acceleration 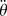 over time. Although the robot recorded the joint angle at 250 Hz, this information was not used due to known issues of numerical noise in the derivation of angular velocity and angular acceleration. Instead, the angular velocity and angular acceleration were derived analytically by taking the first and second derivative of the input command equations describing the angular motion as used for controlling the robot. Next, equation I was used to calculate reference acceleration 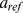. Here, 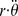 represents the tangential acceleration and 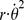 represents the centripetal acceleration, which when taken together as the vector magnitude add up to the overall acceleration of the accelerometer.
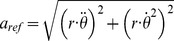
(1)


The average metric output and reference values were calculated over an integer number of oscillating periods in the middle two minutes of each experimental condition (3 minutes), after which absolute and relative measurement errors were expressed. Relative errors were calculated as (Estimated – Reference)/Reference.

For reference purposes, all analyses were repeated based on simulated acceleration signals using the equations as in equation II and equation III. Here, 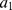 refers to the acceleration signal perpendicular to the length of the bar which captures the tangential acceleration combined with the effect of the gravitational component and 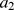 refers to the acceleration signal in parallel to the length of the bar which captures the centripetal acceleration combined with the gravitational component. The centre of rotation is assumed to not change position.
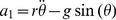
(2)

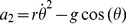
(3)


Metrics ENMO, HFEN, HFEN_+_, BFEN and EN were applied to the raw data collected on the wrist and hip (7 days) after which metric output was averaged over consecutive non-overlapping 1 minute time windows. Further, metrics ENMO, HFEN, HFEN_+_, BFEN and EN were applied to the raw data collected in the human participants where PAEE reference data was available. Here, metric output was averaged per person. A detailed description of the detection of monitor non-wear periods and signal clipping are provided in **Supporting Information S1**. Fifteen minute blocks that were classified as non-wear or clipping were replaced by the average of blocks at the same time periods of the day (from the other days in each individual record). If no data was collected for a certain part of the day then it was imputed by 1 g for metric EN and by 0 g for all other metrics. All signal processing and statistics were performed in R (http://cran.r-project.org).

### Statistics

Means and (relative) differences were computed for the data resulting from the robot experiment. In order to evaluate whether differences between metrics resulted in different measures of free-living human movement, repeated measures ANOVA was used to assess the within- and between-individual explained variance between metrics, stratified by wrist and hip placement. Analyses were performed for all data points excluding non-wear time segments and repeated including imputed data for non-wear time segments. The most important difference is that this would either include or exclude hip accelerometer values for sleeping hours. Results were very similar, and we only report results excluding non-wear time for these analyses. Average and standard deviation of metric output are reported based on imputed data to facilitate the comparison between this study population with future study populations.

For the PAEE analyses, participant inclusion criteria were identical to our previous work [19]: more than 50% detected monitor wear time and at least one day of valid data. Linear regression analysis was used to assess how much of the variation in daily PAEE, expressed in MJ/day, can be explained by each metric in combination with body weight. Additionally, we tested the additive value of metrics by adding combinations of metrics to the regression model.

## Results

**Figure 3:**
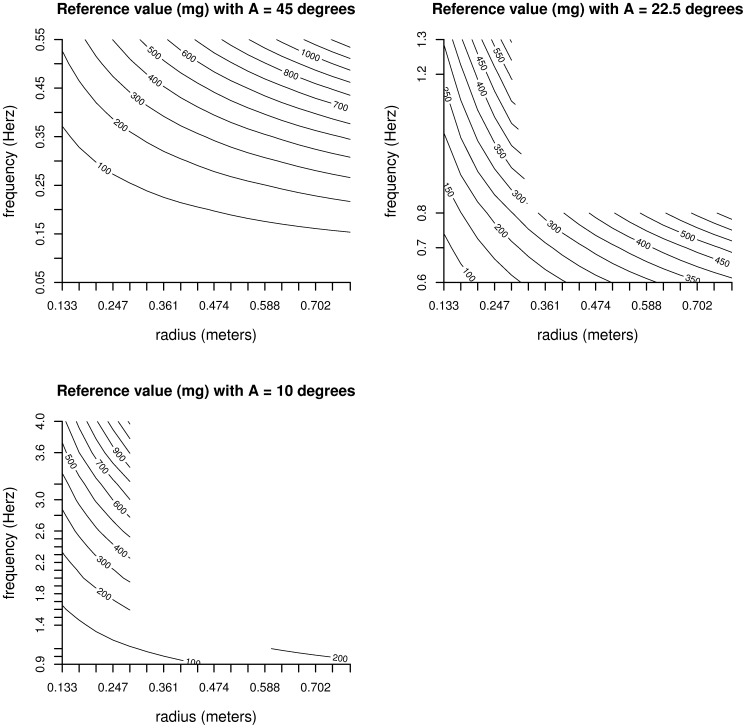
Robot conditions and corresponding reference acceleration (mg), where A = amplitude of angle.

Robot conditions and corresponding reference acceleration are presented in **Figure 3**. The accelerometer attached to the base of joint 5, which in theory should not move, recorded a magnitude of acceleration (vibration) beyond the sensor’s noise level (SD: 2.6 mg = 0.0026 g) for most experimental conditions. On average the acceleration of the robot joint was 4% to 5% of the average acceleration of the accelerometers on the bar attached to the flange, see **Table 2**. The highest value of 76% for ENMO was the result of computed acceleration being close to zero (−5.13 mg).

**Table 2:** Average (mg) and relative (%) acceleration of the base of joint 5 (should ideally be zero) by experimental condition and metric.

		Metrics		
Frequency (Hz)	Angle (°)	ENMO	HFEN	HFEN_+_
0.05–0.2	0–90	−3.9	13.4	9.4
		76.0%	7.1%	6.3%
0.25–0.55	0–90	−4.9	14.2	9.2
		−12.3%	2.2%	2.4%
0.6–0.8	0–45	−2.9	18.9	15.7
		−8.1%	3.7%	3.7%
0.9–1.1	0–20	0.9	21.5	22.0
		6.7%	5.8%	6.4%
1.2–1.3	0–45	1.5	9.3	10.8
		2.0%	1.4%	1.9%
1.4–2.0	0–20	0.1	35.9	35.2
		0.4%	7.8%	7.9%
2.1–3.0	0–20	1.4	17.1	18.3
		0.7%	1.9%	2.1%
3.2–4.0	0–20	2.3	74.8	73.5
		0.2%	4.3%	4.3%
	**Average**	0.7	25.6	24.3
		8.2%	4.3%	4.4%

Relative values are expressed as percentage of average metric output for all accelerometers attached to the bar as fixed to the flange.

The metric output for each accelerometer attached to the bar was compared against the reference acceleration. Metric HFEN_+_ was more accurate compared to metric HFEN with an average difference in absolute measurement error of respectively, 90 mg and 109 mg. Measurement error was lowest for metric HFEN_+_ in all but one experimental conditions based on oscillation frequencies higher than 0.2 Hz. On the contrary, metric ENMO outperformed the other metrics for frequencies of oscillation below 0.2 Hz, see **Table 3**. For all metrics, except ENMO, relative and absolute measurement error was lower for higher radius settings, see **Table 3**.

**Table 3:** Evaluation of metrics using empirically recorded acceleration signals.

Freq.(Hz)	Angle (°)	Radius (m)	Acc. (mg)	ENMO	HFEN	HFEN_+_
0*	0	0.1–0.3	0	−9	4	−5
0*	0	0.3–0.6	0	0	6	6
0*	0	0.6–0.8	0	−3	9	6
0*	22.5	0.1–0.3	0	−4	3	0
0*	22.5	0.3–0.6	0	−11	5	−4
0*	22.5	0.6–0.8	0	−11	7	−4
0.05–0.2	0–90	0.1–0.3	14	−16(−173)	167 (1427)	132 (1184)
0.05–0.2	0–90	0.3–0.6	31	−38 (−162)	155 (619)	112 (447)
0.05–0.2	0–90	0.6–0.8	48	−55 (−144)	152 (442)	107 (343)
0.25–0.55	0–90	0.1–0.3	129	−122 (−98)	435 (498)	212 (272)
0.25–0.55	0–90	0.3–0.6	281	−251 (−93)	364 (194)	89 (76)
0.25–0.55	0–90	0.6–0.8	434	−354 (−86)	308 (108)	−3 (24)
0.6–0.8	0–45	0.1–0.3	161	−153 (−97)	206 (149)	141 (102)
0.6–0.8	0–45	0.3–0.6	351	−328 (−95)	152 (49)	57 (21)
0.6–0.8	0–45	0.6–0.8	541	−465 (−87)	118 (24)	9 (3)
0.9–1.1	0–20	0.1–0.3	134	−128 (−99)	93 (78)	83 (67)
0.9–1.1	0–20	0.3–0.6	293	−292(−100)	73 (27)	44 (17)
0.9–1.1	0–20	0.6–0.8	451	−419 (−93)	68 (16)	35 (8)
1.2–1.3	0–45	0.1–0.3	508	−432 (−87)	160 (35)	63 (14)
1.4–2.0	0–20	0.1–0.3	390	−364 (−95)	72 (22)	54 (16)
2.1–3.0	0–20	0.1–0.3	832	−618 (−79)	47 (7)	22 (3)
3.2–4.0	0–20	0.1–0.3	1700	−779 (−50)	45 (3)	14 (1)

Values are average absolute differences in mg (average relative error % in brackets §) between each metric output and the actual acceleration related to movement for various sections of the experiment.

[Acc, average reference acceleration; *zero movement condition; § Relative measurement error was calculated per experimental condition and then averaged across each section of the experiment].

Replication of the analyses with simulated acceleration signals confirmed the empirical findings as described above. A detailed overview of the results based on simulated acceleration signals are included in **Supporting Information S1**. Data and R-scripts related to the robot experiments are available on our website: http://www.mrc-epid.cam.ac.uk/research/resources.

When metrics were applied to human wrist and hip acceleration signals collected during free-living conditions, repeated measures ANOVA showed that the shared within- and between-individual variances (r-squared) varied between metric pairs and body locations, see **Table 4** **and** **Table 5**. Lowest shared variance was found for metric-pairs involving metric EN; for example, this metric shared 54 and 11% of the within- and between–individual variance, respectively, with metric BFEN for hip acceleration, see **Table 5**. Highest shared variances were observed between the filter-based metrics. For example, metrics HFEN and BFEN as well as versions of HFEN with different cut-off frequencies were all highly correlated both within and between individuals and for both hip and wrist data (r-square values >0.96), see **Table 4** **and** **5**. A difference between wrist and hip worth noting was the shared variance between ENMO and the filter-based metrics HFEN, BFEN and HFEN+. Here, the shared variance within individuals was highest for the hip (0.92 vs. 0.87 on average), while the shared variance between individuals was highest for the wrist (0.87 vs. 0.62 on average), see **Table 4** **and** **Table 5**.

**Table 4:** Explained variance (r^2^) within (above diagonal) and between (below diagonal) individual wrist accelerometer data for all combinations of data processing metrics.

	ω_0_ (Hz)	EN	ENMO	BFEN	HFEN	HFEN	HFEN_+_	HFEN_+_
**ω_0_ (Hz)**		**−**	**−**	*0.2–15*	*0.2*	*0.5*	*0.2*	*0.5*
**EN**	**−**	**−**	0.91	0.61	0.62	0.71	0.75	0.80
**ENMO**	**−**	0.92	**−**	0.80	0.81	0.89	0.91	0.95
**BFEN**	*0.2–15*	0.58	0.80	**−**	0.99	0.96	0.96	0.93
**HFEN**	*0.2*	0.60	0.82	1.00	**−**	0.98	0.97	0.94
**HFEN**	*0.5*	0.64	0.88	0.98	0.99	**−**	0.98	0.98
**HFEN_+_**	*0.2*	0.74	0.91	0.97	0.97	0.98	**−**	0.99
**HFEN_+_**	*0.5*	0.77	0.95	0.94	0.95	0.98	0.99	**−**
*Mean (sd) acceleration [mg]*		1016(9)	32(10)	114(25)	118(26)	93(22)	110(25)	94(23)

[ω_0_: cut-off for frequency filter].

**Table 5:** Explained variance (r^2^) within (above diagonal) and between (below diagonal) individual hip accelerometer data for all combinations of data processing metrics.

	ω_0_ (Hz)	EN	ENMO	BFEN	HFEN	HFEN	HFEN_+_	HFEN_+_
ω_0_ (Hz)		−	−	*0.2–15*	*0.2*	*0.5*	*0.2*	*0.5*
EN	−	−	0.77	0.54	0.55	0.58	0.61	0.63
ENMO	−	0.75	−	0.89	0.90	0.92	0.94	0.95
BFEN	*0.2–15*	0.11	0.46	−	1.00	0.99	0.99	0.98
HFEN	*0.2*	0.10	0.46	1.00	−	0.99	0.99	0.98
HFEN	*0.5*	0.11	0.48	0.98	0.98	−	0.97	0.99
HFEN_+_	*0.2*	0.52	0.85	0.78	0.78	0.75	−	0.99
HFEN_+_	*0.5*	0.54	0.89	0.76	0.75	0.76	0.99	−
*Mean (sd) acceleration [mg]*		1007(15)	18(16)	46(15)	48(15)	42(14)	50(21)	45(20)

[ω_0_: cut-off for frequency filter].

For the modelling of PAEE, HFEN_+_ outperformed metrics ENMO, HFEN, BFEN and EN, explaining 36% of the variance in daily PAEE, see **Table 6**. When pairs of metrics were added to the regression model, no significant additive value was found (p>0.05 corresponding with increases in model r^2^ of less than 0.01).

**Table 6:** Overview of regression models for predicting PAEE (MJ day^−1^) based on N = 63 women.

Model input	ω_0_ (Hz)	SE	R^2^	Equation
EN	−	0.99	0.26*	−56.146 + BW × 0.023 + EN × 57.093
ENMO	−	0.94	0.34**	−0.172 + BW × 0.025 + ENMO × 0.057
BFEN	0.2–15	0.97	0.30**	−0.913 + BW × 0.021 + BFEN × 0.023
HFEN	0.2	0.97	0.30**	−0.905 + BW × 0.021 + HFEN × 0.023
HFEN	0.5	0.95	0.32**	−0.769 + BW × 0.022 + HFEN × 0.027
HFEN_+_	0.2	0.93	0.36**	−1.114 + BW × 0.023 + HFEN_+_ × 0.025
HFEN_+_	0.5	0.93	0.36**	−0.805 +BW × 0.023 + HFEN_+_ × 0.026

[SE: Residual standard error;

** : p<.001;

* : p<.01; ω_0_: cut-off for frequency filter; BW = body weight (kg)].

## Discussion

The present study demonstrates that the choice of signal processing technique for summarising accelerometer data can have a substantial impact on the accuracy with which acceleration related to movement is measured. Subsequently, the choice of signal processing technique impacts on the summary measures of human acceleration data and criterion-related validity for estimating daily PAEE. In the past, physical activity researchers did not have the opportunity to select a metric; the metric decision was made by the manufacturer of the accelerometer [21], [22], [23], [24], [25].

The first and main part of this paper evaluated metrics under a range of standardised kinematic conditions in order to gain insight into how the accuracy of metric output relates to the kinematics of movement. No single metric outperformed all other metrics for all experimental conditions. Metric HFEN_+_ resulted in less measurement error compared to metric HFEN. This result may indicate that HFEN_+_ manages to retrieve some of the non-gravitational acceleration in the lower frequency range and/or remove gravitational acceleration from the frequency range above the filter threshold in contrast to metric HFEN. Metric HFEN_+_ outperformed metrics ENMO and HFEN for the experimental conditions based on oscillating frequencies higher than the cut-off frequency as used by its frequency filter (0.2 Hz), while the ENMO metric outperformed metrics HFEN and HFEN_+_ for experimental conditions based on oscillating frequencies below this cut-off frequency. This difference between HFEN, HFEN_+_ and ENMO may partly be explained by the fact that metrics HFEN and HFEN_+_ aim to remove the gravitational component by making assumptions on its representation in the frequency content of an acceleration signal, while ENMO aims to remove the gravitational component based on assumptions with regard to its magnitude. Metric HFEN_+_ could be seen as a hybrid version of the two approaches as it relies on both an assumption about the representation of gravity in the frequency domain and an assumption about the magnitude of gravity. The mutual assumption by metrics ENMO and HFEN_+_ that gravity is measured as 1 g would not hold true if acceleration sensors are not accurately calibrated and would therefore result in biased metric output. Further, metric ENMO has one additional limitation: For a signal with an offset of 1 g (e.g. containing the gravitational component) and an amplitude of less than 1, taking the square will increase the amplitude. On the contrary, if the square is taken from a signal with no offset (e.g. no gravity) and the amplitude is less than one, then taking the square will decrease the amplitude. Therefore, taking the square of three orthogonal signals like in metric ENMO will result in a stronger contribution of vertical accelerations that alternate around 1 g to the resulting summary measure compared with horizontal accelerations that alternated around 0 g.

The reference acceleration as used for the evaluation of the metrics may not have been exactly equal to the true acceleration that the accelerometers were exposed to; imprecision in accelerometer positioning and system vibrations are possible sources of error. In theory, the acceleration of a rotating and non-translating object is proportional to the distance from its centre of rotation, the radius length. A discrepancy of 5 mm (plausible) in the assessment of accelerometer position would represent 0.6% for the accelerometer farthest away and 3.7% for the accelerometer closest to the axis of rotation. This would translate into a similar degree of error in the calculated reference acceleration (0.6–3.7%). Secondly, vibrations of the whole robot during operation may have resulted in the true acceleration exposure being higher than what we calculated it to be. The accelerometer attached to the base of joint 5 did record acceleration beyond the sensor’s noise level likely resulting from the movement of the robot system itself. We believe that robot movement was caused by the supporting frame that vibrated towards the extreme experimental conditions; the robot itself has a high stiffness. The accelerometers attached to the bar mounted on the flange have been exposed to these vibrations as well as those intended by the experimental design. The replication of the robot analyses with simulated acceleration signals confirmed the empirical findings, indicating that environmental vibrations had no significant impact.

As for the analyses conducted on data collected during human daily life, the shared within-individual variances were all above 80% between metrics which make some attempt at removing the gravitational component, indicating the pattern within an individual is picked up quite similarly between those metrics. The between-individual shared variances, which is a measure of the metrics’ ability to rank individuals similarly, showed some differences between hip and wrist positions, most notably lower similarity between ENMO and frequency-filtered metrics for hip than wrist. Whether this reflects differences in monitoring protocols (24-hr vs. non-sleep time), differences in signal to error ratio and/or differences in frequency characteristics of the gravitational component as measured by triaxial accelerometry at these two positions is difficult to conclude from our data. However, it should be noted that shared variances only indicate to what extent metrics are similar in describing variance on a relative level but not what the shared variance represents; it will also include any correlated measurement error and should therefore be interpreted with caution.

Physical activity-related energy expenditure and body acceleration are only distally related to each other. As a consequence, differences in explained variance in daily PAEE does not serve as direct evidence for a metric’s ability to remove the gravitational component.

HFEN_+_ outperformed HFEN when using daily PAEE as a reference, which confirms the findings from the higher frequency conditions in the robot experiment. Further, ENMO turned out to be a good alternative for HFEN_+_. The correspondence between the strong performance of ENMO in explaining variance in PAEE in the current analysis with the strong performance of ENMO in the lower frequency range of the robot experiment might indicate that wrist acceleration in daily life is dominated by translational accelerations and/or accelerations resulting from low frequency rotations. A second explanation for the strong performance of metric ENMO may be its higher sensitivity to vertical accelerations (vertical acceleration is amplified) as explained above. The latter would indicate that vertical wrist accelerations are the stronger determinant of daily PAEE compared with accelerations in the horizontal plane. A third and final explanation could be that ENMO is more accurate at measuring translational acceleration compared with some of the other metrics, as the signal is never deformed by frequency filtering in ENMO.

The subtraction of one in ENMO has a constant effect on all the metric output and would in theory be perfectly correlated with EN, which should therefore correlate the same with PAEE. However, there is one additional difference between the two metrics, namely the replacement of negative values by zero in ENMO, which explains why metric ENMO outperforms metric EN for the prediction of PAEE. The truncation of negative values to zero could be hypothesized to be an effective correction mechanism for errors in the subtraction of the gravitational component.

Filter settings for HFEN and HFEN_+_ were briefly evaluated indicating that a 0.5 Hz filter cut-off frequency may perform slightly better than a 0.2 Hz filter cut-off frequency for predicting PAEE. A more thorough optimization of filter settings could lead to further improvement but also introduces the risk of over-fitting filter configurations to one study population, which may not generalise to others.

One previous study investigated the need for removing the gravitational component using metabolic energy expenditure as reference method and concluded that attempting to remove the gravitational component is not worth the effort [13]. In that particular study, body segment position and orientation over time were derived from a 2D optical system and used to simulate acceleration sensor output [13]. The validity of these simulations was only assessed for the lower back position and not for the five other simulated sensor positions, complicating the interpretation of study results. Our own results indicate that attempting to remove the gravitational component is worth the effort for estimating daily PAEE in humans based on wrist accelerometry as ENMO, HFEN and HFEN_+_ clearly outperformed metric EN.

Additional research is needed to explore the potential of combining metrics in a fashion that the best metric is chosen depending on the kinematic conditions. It should be noted that all PAEE-related results apply to the wrist placement and cannot be generalized to other body locations. Future research is therefore also needed to explore the importance of metric selection for other body locations, in particular commonly used positions at the lower back and the hip.

### Conclusions

In conclusion, none of the metrics as evaluated systematically outperformed all other metrics across a wide range of standardised kinematic conditions. However, choice of metric explains different degrees of variance in daily physical activity.

## Supporting Information

Supporting Information S1 **Additional information on signal processing and replication of robot findings with simulated data.**
(DOC)

## References

[1] Corder K, Ekelund U, Steele RM, Wareham NJ, Brage S (2008) Assessment of physical activity in youth. J Appl Physiol 105: 977–987. 18635884 10.1152/japplphysiol.00094.2008

[2] Wareham NJ, Rennie KL (1998) The assessment of physical activity in individuals and populations: why try to be more precise about how physical activity is assessed? Int J Obes Relat Metab Disord 22 Suppl 2S30–38. 9778094

[3] Wong MY, Day NE, Luan JA, Chan KP, Wareham NJ (2003) The detection of gene-environment interaction for continuous traits: should we deal with measurement error by bigger studies or better measurement? Int J Epidemiol 32: 51–57. 12690008 10.1093/ije/dyg002

[4] Hagstromer M, Troiano RP, Sjostrom M, Berrigan D (2010) Levels and patterns of objectively assessed physical activity–a comparison between Sweden and the United States. Am J Epidemiol 171: 1055–1064. 20406758 10.1093/aje/kwq069

[5] Colley RC, Garriguet D, Janssen I, Craig CL, Clarke J, et al (2010) Physical activity of Canadian children and youth: accelerometer results from the 2007 to 2009 Canadian Health Measures Survey. Health Rep 22: 15–23. 21510586

[6] Brandes M, Zijlstra W, Heikens S, van Lummel R, Rosenbaum D (2006) Accelerometry based assessment of gait parameters in children. Gait Posture 24: 482–486. 16427287 10.1016/j.gaitpost.2005.12.006

[7] Moe-Nilssen R, Helbostad JL (2004) Estimation of gait cycle characteristics by trunk accelerometry. J Biomech 37: 121–126. 14672575 10.1016/s0021-9290(03)00233-1

[8] Aminian K, Robert P, Buchser EE, Rutschmann B, Hayoz D, et al (1999) Physical activity monitoring based on accelerometry: validation and comparison with video observation. Med Biol Eng Comput 37: 304–308. 10505379 10.1007/BF02513304

[9] Veltink PH, Bussmann HB, de Vries W, Martens WL, Van Lummel RC (1996) Detection of static and dynamic activities using uniaxial accelerometers. IEEE Trans Rehabil Eng 4: 375–385. 8973963 10.1109/86.547939

[10] Redmond DP, Hegge FW (1985) Observations on the design and specification of a wrist-worn human activity monitoring system. Behavior Research Methods 17: 11.

[11] Van Someren EJ, Lazeron RH, Vonk BF, Mirmiran M, Swaab DF (1996) Gravitational artefact in frequency spectra of movement acceleration: implications for actigraphy in young and elderly subjects. J Neurosci Methods 65: 55–62. 8815309 10.1016/0165-0270(95)00146-8

[12] Bourke AK, O'Donovan K, Clifford A, G OL, Nelson J. Optimum gravity vector and vertical acceleration estimation using a tri-axial accelerometer for falls and normal activities; Conf Proc IEEE Eng Med Biol Soc; 2011; Boston, Massachusetts, USA. 7896–7899. 10.1109/IEMBS.2011.6091947 22256171

[13] Bouten CV, Sauren AA, Verduin M, Janssen JD (1997) Effects of placement and orientation of body-fixed accelerometers on the assessment of energy expenditure during walking. Med Biol Eng Comput 35: 50–56. 9136191 10.1007/BF02510392

[14] Roetenberg D, Slycke PJ, Veltink PH (2007) Ambulatory Position and Orientation Tracking Fusing Magnetic and Inertial Sensing. IEEE Transactions on Biomedical Engineering 54: 883–890. 17518285 10.1109/TBME.2006.889184

[15] Sabatini AM (2006) Quaternion-Based Extended Kalman Filter for Determining Orientation by Inertial and Magnetic Sensing. IEEE Transactions on Biomedical Engineering 53: 1346–1356. 16830938 10.1109/TBME.2006.875664

[16] Yun X, Lizarraga M, Bachmann ER, McGhee RB. An Improved Quaternion-Based Kalman Filter for Real-Time Tracking of Rigid Body Orientation; Intelligent robots and systems; 2003 IEEE/RSJ international conference on intelligent robots and systems (IROS 2003); 2003, Oct; Las Vegas, NV. IEEE. 1074–1079.

[17] Piazzi A, Visioli A. An interval algorithm for minimum-jerk trajectory planning of robot manipulators; Decision and control; 1997, Dec; San Diego, CA. IEEE. 1924–1927.

[18] Kyriakopoulos KJ, Saridis GN. Minimum jerk path generation; IEEE International Conference on Robotics and Automation; 1988, 24–29 Apr Philadelphia, PA, USA.

[19] van Hees VT, Renstrom F, Wright A, Gradmark A, Catt M, et al (2011) Estimation of daily energy expenditure in pregnant and non-pregnant women using a wrist-worn tri-axial accelerometer. PLoS One 6: e22922. 21829556 10.1371/journal.pone.0022922 PMC3146494

[20] van Hees VT, Pias M, Taherian S, Ekelund U, Brage S. A method to compare new and traditional accelerometry data in physical activity monitoring; 2nd IEEE International WoWMoM Workshop on Interdisciplinary Research on E-Health Services and Systems (IREHSS); 2010, 14–17 June; Montreal, Canada. 1–6.

[21] Plasqui G, Joosen AM, Kester AD, Goris AH, Westerterp KR (2005) Measuring free-living energy expenditure and physical activity with triaxial accelerometry. Obes Res 13: 1363–1369. 16129718 10.1038/oby.2005.165

[22] Bonomi AG, Plasqui G, Goris AH, Westerterp KR (2010) Estimation of free-living energy expenditure using a novel activity monitor designed to minimize obtrusiveness. Obesity (Silver Spring) 18: 1845–1851. 20186133 10.1038/oby.2010.34

[23] Corder K, Brage S, Wright A, Ramachandran A, Snehalatha C, et al (2011) Physical activity energy expenditure of adolescents in India. Obesity (Silver Spring) 18: 2212–2219. 10.1038/oby.2010.4 20134412

[24] Assah FK, Ekelund U, Brage S, Corder K, Wright A, et al (2009) Predicting physical activity energy expenditure using accelerometry in adults from sub-Sahara Africa. Obesity (Silver Spring) 17: 1588–1595. 19247268 10.1038/oby.2009.39 PMC2771276

[25] Rothney MP, Brychta RJ, Meade NN, Chen KY, Buchowski MS (2010) Validation of the ActiGraph two-regression model for predicting energy expenditure. Med Sci Sports Exerc 42: 1785–1792. 20142778 10.1249/MSS.0b013e3181d5a984 PMC2919650

