# Supplementary material for: Separating Movement and Gravity Components in an Acceleration Signal and Implications for the Assessment of Human Daily Physical Activity
Source: PLoS One. 2013 Apr 23;8(4):e61691. doi: 10.1371/journal.pone.0061691 (PMC3634007; doi:10.1371/journal.pone.0061691)
Supplement: Supporting Information S1 — Additional information on signal processing and replication of robot findings with simulated data. (DOC) [file pone.0061691.s001.doc]

**Supporting Information S1**

**Motivation for metric HFEN_+_**

If there is no rotational movement the Euclidean norm of the low-frequency components (LFEN) must be equal to the magnitude of gravity (= 1 g). During rotational movement, however, normal (centrifugal) acceleration and/or the tangential acceleration will be added to the signal. The frequency content of the angular velocity and the cut-off frequency as set for LFEN will then determine whether the accelerations related to rotation are added to the high- or the low-frequency content of the signal. At the same time acceleration related to gravity, which in the non-rotational case was fully present in the low frequency range of the signal, may have moved to the high frequency range as a result of the rotation which may be at a frequency above the cut-off frequency as set for LFEN; this will cause the value of LFEN to drop below 1 g. The difference between LFEN and “1 g” may therefore be an indicator for measurement error in HFEN. This idea was initially verified in a simple pilot experiment, in which one tri-axial accelerometer was attached to a horizontal shaker machine and shaken (but not rotated) at various frequencies (range: 0.5 - 4 Hz), secondly the accelerometer was attached to the rotating wheel of a treadmill and spun at various frequencies (range: 0.5 - 4 Hz). **Figure 1** shows how LFEN and HFEN, respectively, are affected by translation and rotation at increasing frequencies.


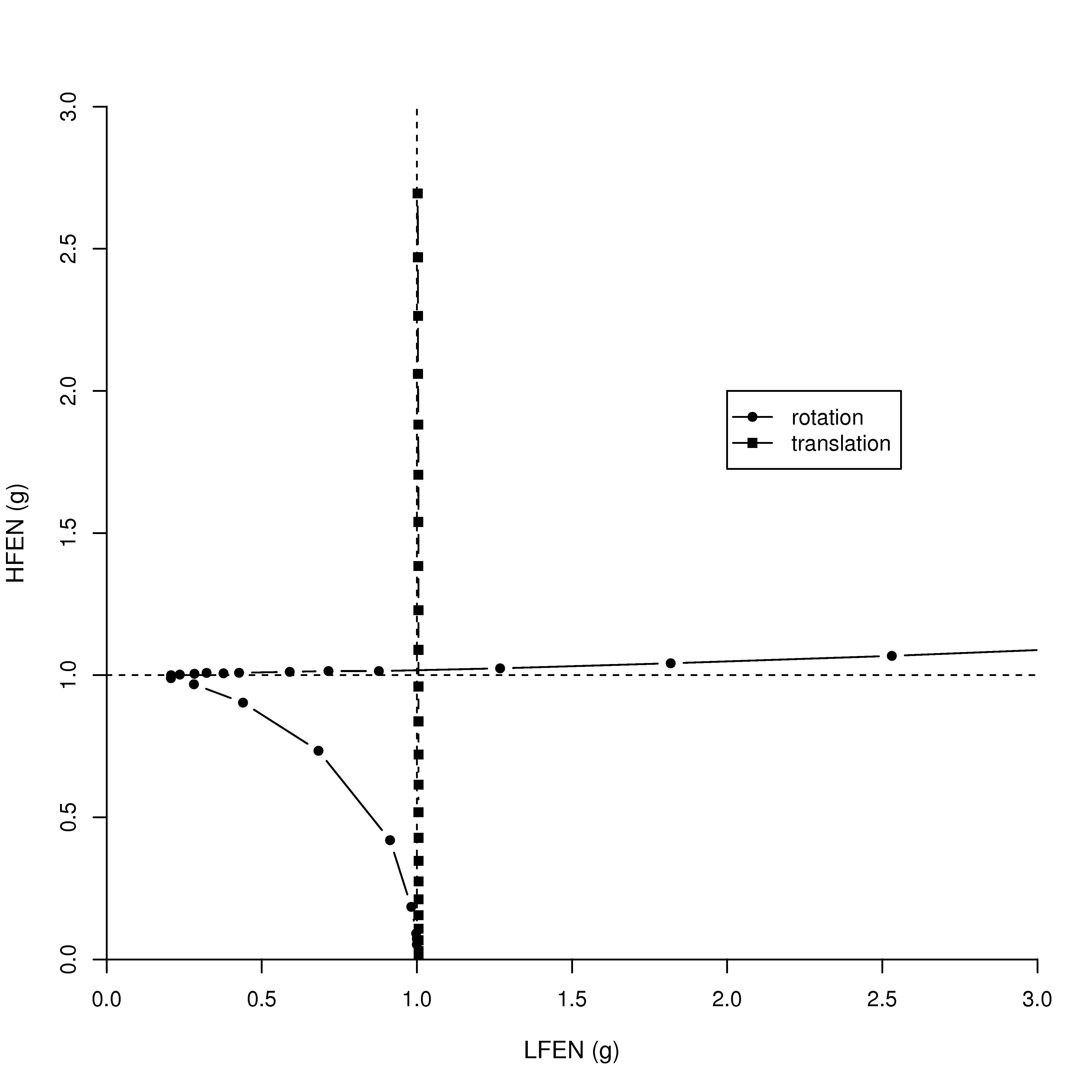


Figure 1: Pilot experiment to demonstrate the idea underlying the fourth and fifth metric, respectively HFEN_+_

# Replication of robot findings based on simulated acceleration signals

In order to verify the correctness of the empirical findings all robot-based analysis were replicated based on simulated acceleration signals. The corresponding results are presented in the table below and confirm the empirical findings as described in the paper to which this document is a supplement. The minor differences may be explained by vibration of the supporting table or vibration of the bar that holds the accelerometers. An alternative explanation for the minor differences between simulated and empirical results could be sensor characteristics, e.g. hysteresis or crosstalk between sensors at high angular velocities.

**Table S1: Evaluation of metric using simulated acceleration signals. Average absolute difference in mg (average relative error % in brackets §) between each metric output and the actual acceleration related to movement for various sections of the experiment.**

| **Freq. (Hz)** | **Angle (º)** | **Radius (m)** | **Acc. (mg)** | **ENMO** | **HFEN** | **HFEN+** |
| --- | --- | --- | --- | --- | --- | --- |
| 0.05-0.2 | 0 – 90 | 0.1 – 0.3 | 14 | -15 (-99) | 175 (1573) | 75 (270) |
| 0.05-0.2 | 0 – 90 | 0.3 – 0.6 | 31 | -33 (-99) | 160 (651) | 59 (69) |
| 0.05-0.2 | 0 – 90 | 0.6 – 0.8 | 48 | -50 (-98) | 146 (377) | 43 (11) |
| 0.25-0.55 | 0 – 90 | 0.1 – 0.3 | 129 | -124 (-95) | 427 (470) | 151 (201) |
| 0.25-0.55 | 0 – 90 | 0.3 – 0.6 | 281 | -251 (-89) | 355 (182) | 29 (45) |
| 0.25-0.55 | 0 – 90 | 0.6 – 0.8 | 434 | -355 (-83) | 300 (101) | -74 (3) |
| 0.6-0.8 | 0 – 45 | 0.1 – 0.3 | 161 | -154 (-94) | 214 (151) | 76 (58) |
| 0.6-0.8 | 0 – 45 | 0.3 – 0.6 | 351 | -309 (-88) | 166 (52) | -3 (2) |
| 0.6-0.8 | 0 – 45 | 0.6 – 0.8 | 541 | -438 (-81) | 130 (25) | -69 (-12) |
| 0.9-1.1 | 0 – 20 | 0.1 – 0.3 | 134 | -130 (-95) | 100 (83) | 14 (14) |
| 0.9-1.1 | 0 – 20 | 0.3 – 0.6 | 293 | -267 (-90) | 78 (28) | -19 (-6) |
| 0.9-1.1 | 0 – 20 | 0.6 – 0.8 | 451 | -389 (-85) | 62 (14) | -45 (-10) |
| 1.2-1.3 | 0 – 45 | 0.1 – 0.3 | 508 | -413 (-82) | 182 (40) | -11 (0) |
| 1.4-2.0 | 0 – 20 | 0.1 – 0.3 | 390 | -335 (-91) | 74 (24) | -21 (-5) |
| 2.1-3.0 | 0 – 20 | 0.1 – 0.3 | 832 | -640 (-81) | 31 (5) | -57 (-7) |
| 3.2-4.0 | 0 – 20 | 0.1 – 0.3 | 1700 | -798 (-50) | 19 (1) | -68 (-4) |

[Acc, average reference acceleration; § Relative measurement error was calculated per experimental condition and then averaged across each section of the experiment].

**Additional explanation on signal processing in real life data**

*Procedure for non-wear detection*

The procedure for non-wear detection was modified in comparison to the procedure as applied in our previous publication [1]. Instead of 30-minute time windows 60-minute time windows were used to decrease the chance of accidently detecting short sedentary periods as non-wear time. The windows were overlapping (15 minute steps, window overlap of 45 minutes), which was done to improve the accuracy of detecting the boundaries of non-wear time as opposed to non-overlapping time windows.

Inspection of unpublished data on non-wear classification by the algorithm as described in our published work [1] indicated that the algorithm does not cope well with periods of monitor transportation per post. Here, long periods of non-wear are briefly interrupted by periods of movement, which are normally interpreted as monitor wear. Therefore, the algorithm was expanded with an additional stage in which the plausibility of “wear-periods” in-between non-wear periods is tested. Short periods of detected wear-time in-between longer periods of detected non-wear were classified as non-wear time based on the duration and the proportion of the duration relative to the bordering periods of detected non-wear-periods. The following criteria were derived from visual observation of various datasets using knowledge about study protocols. All detected wear-periods of less than six hours and less than 30% of the combined duration of their bordering non-wear periods were classified as non-wear. Additionally, all wear-periods of less than three hours and which formed less than 80% of their bordering non-wear periods were classified as non-wear. The motivation for selecting a relatively high criteria (< 30%) in combination with a long period (6hrs) and a low criteria (< 80%) in combination with a short period (3 hrs) was that long period are more likely to be actually related to monitor wear time. A visual model was created, see **Figure 2**. Here, units of time are presented in squares and marked grey if detected as non-wear time. Period C is detected as wear-time and borders to non-wear periods B and D, see **Figure 2**. If the length of C is less than six hours and C divided by the sum of B and D is less than 0.3 then the first criteria is met and block C is turned into a non-wear period.


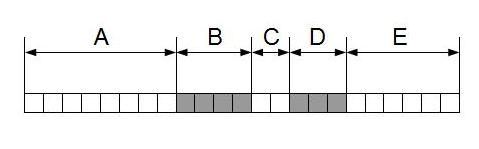


Figure 2: Visual model of periods in time classified as wear (white) or non-wear (grey)

By visual inspection of >100 traces from a large observational study it turned out that applying this stage in three iterative stages allowed for improved classification of periods characterised by intermittent periods of non-wear and apparent wear. Further, an additional rule was introduced for the final 24 hours of each measurement. The final 24 hours are often considered the period in which the accelerometer is potentially taken off but moved because of transportation, e.g. by the mail service. All wear-periods in the final 24 hrs of each measurement shorter than three hours and preceded by at least one hour of non-wear time were classified as non-wear. Finally, if the measurement starts or ends with a period of less than three hours of wear followed by non-wear (any length) then this period of wear is classified as non-wear. These additional criteria for screening the beginning and end of the accelerometer file reflect the likelihood of movements that are involved when starting the accelerometer or downloading the data from the accelerometer.

*Detection of clipping and locking*

The data quality check was expanded with a section in which the acceleration signal was screened for ‘clipping’. If more than 50% of the data points in a 15 minute time window were higher than 5.5g (close to the maximal dynamic range of this sensor) the corresponding time period was considered as potentially corrupt data, which may be explained by the sensor getting stuck at its extreme value.

**Data availability for free-living component**

The analysis commenced with 99 participants which is identical to the analysis as described in our published work [1]. However, as a result of modifications to the algorithm for non-wear detection, six instead of two participants were excluded because they had less than one valid day of data, resulting in 63 non-pregnant women and 30 pregnant women of which we only used the non-pregnant women for the analysis as described in the paper. The criteria of 50% ‘wear time’ did not result in additional participant exclusion.

One accelerometer recording was discovered to have accidently been set up to record data at a sample frequency of 80Hz instead of 40 Hz, which was not discovered or accounted for in the published results [1]. The data file was not excluded from further analyses but corrections were made to signal processing in the current study to account for this.

**Literature list**

[1] van Hees VT, Renstrom F, Wright A, Gradmark A, Catt M, Chen KY, et al. Estimation of daily energy expenditure in pregnant and non-pregnant women using a wrist-worn tri-axial accelerometer. PloS one. 2011;6(7):e22922.
